# Effect of add-on hydroxychloroquine therapy on serum proinflammatory cytokine levels in patients with systemic lupus erythematosus

**DOI:** 10.1038/s41598-022-14571-6

**Published:** 2022-06-17

**Authors:** Risa Wakiya, Kiyo Ueeda, Shusaku Nakashima, Hiromi Shimada, Tomohiro Kameda, Mai Mahmoud Fahmy Mansour, Mikiya Kato, Taichi Miyagi, Koichi Sugihara, Mao Mizusaki, Rina Mino, Norimitsu Kadowaki, Hiroaki Dobashi

**Affiliations:** Division of Hematology, Rheumatology and Respiratory Medicine, Department of Internal Medicine, Faculty of Medicine, Kagawa University, 1750-1 Ikenobe, Miki-cho, Kita-gun, Kagawa 761-0793 Japan

**Keywords:** Systemic lupus erythematosus, Cytokines, Interleukins, Tumour-necrosis factors

## Abstract

We investigated the effect of hydroxychloroquine (HCQ) as an add-on treatment to immunosuppressants on the expression of proinflammatory cytokines in patients with systemic lupus erythematosus. Serum levels of tumor necrosis factor (TNF)-α, interleukin (IL)-2, IL-6, IL-8, vascular endothelial growth factor (VEGF)-A, monocyte chemotactic protein-1 (MCP-1), macrophage inflammatory protein-1α (MIP-1α), and interleukin 1 receptor antagonist (IL-1ra) were measured immediately before and 3 months after treatment with oral HCQ. Among the 51 patients enrolled in the study, HCQ treatment led to significantly reduced serum levels of TNF-α, IL-6, IL-8, VEGF-A, IL-1ra, and IL-2 (p < 0.0001; p = 0.0006; p = 0.0460, p = 0.0177; p < 0.0001; p = 0.0282, respectively) and to decreased (but not significantly) levels of MIP-1α (p = 0.0746). No significant changes were observed in the serum MCP-1 levels before and after HCQ administration (p = 0.1402). Our results suggest that an add-on HCQ treatment modulates the expression of proinflammatory cytokines even in systemic lupus erythematosus patients with low disease activity.

## Introduction

Systemic lupus erythematosus (SLE) is a chronic systemic autoimmune disease in which multiple organ systems can be damaged by autoantibodies, immune complexes, and inflammation^1^. The pathogenesis of SLE involves a complex interplay of immunological, genetic, and environmental factors^2^. Several studies have identified associations between levels of several proinflammatory cytokines and SLE disease activity with specific clinical manifestations^3–7^. The levels of interferon (IFN) and IFN-inducible chemokines, such as macrophage inflammatory protein-1 (MIP-1), monocyte chemotactic protein-1 (MCP-1), and interferon-inducible protein-10, are reported to correlate with disease activity as measured by disease activity indices such as the erythrocyte sedimentation rate and anti-dsDNA antibody titer^3^. Other proinflammatory cytokines shown to correlate with SLE disease activity include tumor necrosis factor-α (TNF-α), interleukin (IL)-6, IL-8, IL-10, and vascular endothelial growth factor (VEGF)^4–7^.

The European League Against Rheumatism recommended hydroxychloroquine (HCQ) treatment for all patients with SLE in 2019^8^. HCQ was approved for the treatment of SLE in Japan in July 2015; since then, it has been prescribed as an add-on treatment for many SLE patients on immunosuppressants, particularly for women of child-bearing age or patients with refractory rash. Because multiple beneficial effects of HCQ for SLE are reported^9^, additional HCQ treatment may be given to SLE patients with low disease activity (LDA) despite the lack of information regarding the mechanism of action of HCQ.

HCQ therapy has been reported to have a modulatory effect on interferon in patients with SLE^10–13^. However, there are few reports on the effects of HCQ on inflammatory cytokines, and little is known about the effects on biomarkers when HCQ is additionally administered to patients with SLE who are maintaining LDA with conventional therapy. In the present study, we investigated the effect of add-on HCQ on the levels of proinflammatory cytokines in SLE patients with LDA who were receiving immunosuppressants.

## Results

### Baseline characteristics and serum levels of proinflammatory factors

Patients who added immunosuppressant therapy after starting HCQ administration and patients who discontinued HCQ administration within 3 months were excluded from this study. Fifty-one SLE patients (47 women and 4 men) with sustained LDA of at least 3 months duration were enrolled in this study. The cohort baseline characteristics are shown in Table 1. The mean age [± standard deviation (SD)] was 42.1 ± 13.0 years. The mean disease duration was 15.4 ± 11.4 years, and the median Safety of Estrogens in Lupus Erythematosus National Assessment SLE Disease Activity Index (SELENA-SLEDAI) score was 4. Of the 51 patients, 30 had active skin involvement, and the median Cutaneous Lupus Erythematous Disease Area and Severity Index (CLASI) activity score was 3.

**Table 1:** Baseline characteristics of SLE patients enrolled in the study (n = 51).

Characteristics	n = 51
Female, n (%)	47 (92)
Age, years, mean ± SD	42.1 ± 13.0
Disease duration, years, mean ± SD	15.4 ± 11.4
Past involvement	
Renal involvement	22 (43)
Duration of CR free, years	6.1 ± 5.2
Complication of APS	9 (18)
**Disease activity**	
SELENA-SLEDAI score, Median (range)	4 (0–8)
Current skin involvement, %	30 (59)
CLASI activity score	3 (0–14) (n = 30)
CLASI damage score	0 (0–5) (n = 30)
Anti-dsDNA positive, n (%)*^1^	18 (36)
Anti-dsDNA (IU/mL)	5.4 (5–82.8)
C3 (mg/dL)	76 (40–150)
C4 (mg/dL)	13 (2–33)
CH50 (U/mL)	34.1 (14–57.5)
Low complement, n (%)*^2^	27 (53)
White blood cells (/μL)	5000 (1460–10,000)
Lymphocytes (/μL)	1171 (349–3786)
Platelet (× 10^4^/μL)	21.8 (6.7–40.2)
**Concomitant immunosuppressive treatments**	
Prednisone	
n (%)	44 (86)
Median dosage, mg/day (range)	5.0 (1–10)
Other immunosuppressants*^3^	27 (53)
Tacrolimus	15 (29)
Mycophenolate mofetil	8 (16)
Cyclosporine A	2 (4)
Azathioprine	2 (4)
Mizoribine	2 (4)
Methotrexate	1 (2)

*^1^Anti-dsDNA positive is defined as anti-dsDNA titer over 12 IU/mL.

*^2^Low complement is defined as C3, C4, and CH50 concentrations less than 68 mg/dL, less 12 mg/dL, and 30 U/mL, respectively.

*^3^Three patients received multiple immunosuppressants.

*APS* anti-phospholipid antibody syndrome, *NPSLE* neuropsychiatric systemic lupus erythematosus.

Serum levels of TNF-α, IL-6, IL-8, MCP-1, VEGF-A, and IL-1ra at baseline were significantly higher in SLE patients without skin involvement than in those with skin involvement (Table 2). Serum MCP-1 levels correlated with the SLEDAI score (r = 0.30; *p* = 0.0339). Serum MCP-1 and IL-1ra levels tended to be higher in SLE patients with anti-dsDNA antibody positive than in those with DNA antibody negative. However, no significant differences were observed between serum levels of complement factors or dsDNA antibodies in the baseline levels of all proinflammatory cytokines measured in this study. Serum IFN-α was detected in only 6 of the 51 patients (date not shown).

**Table 2:** Association between clinical parameters and proinflammatory cytokines at baseline.

	Anti-dsDNA positive			Low complement			Skin involvement		
	+ (n = 18)	− (n = 33)	P	+ (n = 27)	− (n = 24)	P	+ (n = 30)	− (n = 21)	P
**Concentration**									
TNF-α, pg/mL	4.2 (2.4, 13.3)	3.7 (2.1, 7.1)	0.3567	4.2 (3.0, 6.8)	2.9 (1.6, 8.7)	0.3443	3.4 (1.6, 5.3)	4.6 (2.9, 13.3)	0.0459*
IL-6, pg/mL	2.1 (1.4, 6.0)	1.6 (1.2, 3.3)	0.2739	1.8 (1.4, 4.7)	1.6 (1.1, 2.9)	0.2948	1.4 (1.0, 3.2)	2.0 (1.5, 38.9)	0.0200*
IL-8, pg/mL	9.9 (1.8, 49.5)	3.7 (1.9, 9.3)	0.2038	4.7 (1.9, 11.3)	7.6 (1.8, 61.9)	0.4593	2.6 (1.5, 5.7)	10.8 (6.4, 80.8)	0.0003*
MCP-1, pg/mL	267.9 (179.4. 493.2)	193.8 (149.7, 293.2)	0.0653	216.5 169.6, 354.0)	227.1 (144.5, 317.2)	0.1713	167.2 (147.5, 260.2)	277.0 (233.6, 355.1)	0.0029*
VEGF-A, pg/mL	63.3 (48.7, 84.8)	42.6 (29.2, 73.8)	0.1104	51.0 (34.5, 92.8)	54.1 (30.9, 67.2)	0.7699	41.8 (26.8, 60.4)	78.8 (51.1, 99.2)	0.0015*
IL-1ra, pg/mL	1124.0 (557.6, 2241.5)	653.2 (463.6, 1041.5)	0.0713	688.7 (534.5, 1205.3)	694.4 (420.9, 1509.8)	0.6438	636.5 (466.8, 983.5)	1092.4 (649.3, 2091.3)	0.0594*
MIP-1a, pg/mL	128.2 (104.6, 228.2)	124.5 (116.7, 163.2)	0.5292	120.6 (95.2, 164.6)	137.9 (120.6, 176.1)	0.1959	124.5 (120.6, 137.3)	152.1 (95.0, 1093.8)	0.6004
IL-2, pg/mL	8.3 (4.8, 13.2)	9.7 (4.3, 10.5)	0.6951	9.7 (4.5, 10.2)	9.0 (4.5, 10.7)	0.8551	10.0 (6.8, 10.4)	4.9 (4.4, 27.7)	0.2434

Median (25% quantile, 75% quantile).

*p < 0.05, Wilcoxon rank sum test.

### Serum levels of proinflammatory factor after HCQ treatment

**Figure 1:**
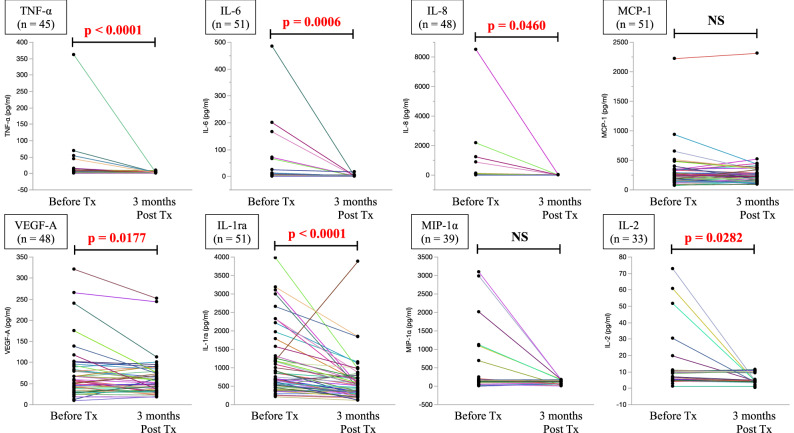
Serum cytokine levels before and after hydroxychloroquine treatment. Serum levels of the indicated cytokines and factors were measured before and after 3 months (3 M Post) of treatment (Tx) with hydroxychloroquine. Colored lines represent individual patients. *p* values were determined using the Wilcoxon signed-rank test. *NS* not significant.

We analyzed serum levels of proinflammatory cytokines at 3 months after initiation of HCQ treatment. As shown in Fig. 1, TNF-α, IL-6, IL-8, VEGF-A, IL-1ra, and IL-2 decreased significantly after HCQ treatment. Although MIP-1α also decreased, the difference from baseline was not significant. In the patients with MCP-1 expression at baseline (n = 51), no change was observed after the addition of HCQ treatment (Fig. 1). No associations were observed between changes in serum proinflammatory cytokine levels and improvements in immunological biomarkers or disease activity scores (Table 3).

**Table 3:** Association between clinical parameters and changes in proinflammatory cytokines.

	Change in anti-dsDNA antibody titer, IU/mL		Change in C3 level, mg/dL		Change in C4 level, mg/dL		Change in CH50 level, U/mL		CLASI responder^¶^ (n = 21)	CLASI nonresponder^¶^ (n = 9)	P*^2^
	ρ	P*^1^	ρ	P*^1^	ρ	P*^1^	ρ	P*^1^			
TNF-α, pg/mL	0.088	0.5658	− 0.17	0.2609	0.011	0.9440	0.042	0.7866	− 0.7 (− 1.4, − 0.5)	− 0.01 (− 1.4, 0.2)	0.1357
IL-6, pg/mL	0.059	0.6800	− 0.10	0.4719	0.044	0.7570	0.074	0.6037	− 0.2 (− 0.9, 0.2)	− 0.1 (− 1.4, 0. 5)	0.5563
IL-8, pg/mL	0.19	0.1966	0.035	0.8119	0.093	0.5279	0.033	0.8255	0.08 (− 0.9, 1.4)	− 0.2 (− 1.4, 1.6)	0.7306
MCP-1, pg/mL	− 0.068	0.6332	0.068	0.6350	− 0.12	0.4034	0.050	0.7303	− 16.2 (− 31.1, 64.9)	− 18.1 (− 84.8, 16.5)	0.2579
VEGF-A, pg/mL	0.22	0.1248	− 0.037	0.8024	0.12	0.4041	0.15	0.3073	− 0.6 (− 14.3, 9.1)	− 1.3 (− 18.0, 12.5)	0.8262
IL-1ra, pg/mL	− 0.0078	0.9567	− 0.058	0.6859	0.0097	0.9460	0.13	0.3592	− 150.9 (− 517.5, − 40.7)	− 289.9 (− 563.6, − 195.4)	0.2393
MIP-1a, pg/mL	0.19	0.2532	− 0.044	0.7904	0.16	0.3189	0.076	0.6444	− 1.8 (− 3.9, 3.9)	− 64.5 (− 144.6, 15.7)	0.9495
IL-2, pg/mL	0.29	0.1063	0.096	0.5954	0.29	0.1074	− 0.0029	0.9871	0.3 (− 0.6, 0.8)	− 0.8 (− 2.3, 1.2)	0.5383

The association between the changes in proinflammatory cytokines and changes in anti-dsDNA titer levels and complement levels was analyzed using univariate analysis. ρ indicates Spearman’s correlation coefficient. *^1^p < 0.05, Spearman’s correlation.

^¶^ Of the patients with skin lesion, 21 cases met the criteria for CLASI responder ( +) and 9 cases did not ( −). The median (25% quantile–75% quantile) of change in each proinflammatory cytokine is shown. *^2^ p < 0.05, Wilcoxon rank sum test.

### Safety

The major adverse events observed during the 3-month period were diarrhea in 12 patients (23.5%) and rash in 4 patients (7.8%). Other adverse events included fatigue, fever, and eye symptoms in two patients each. Regarding ocular symptoms, color blindness and blurred vision were observed in one patient each; however, no ophthalmologic abnormality was noted in either case, and the symptoms improved within a few days. For four patients who had diarrhea and two patients who had rashes, the HCQ dose was reduced; however, it was noted that the adverse events improved spontaneously in other cases as well without HCQ dose reduction.

## Discussion

Previous studies have demonstrated significant associations between proinflammatory cytokines, especially IL-1, IL-6, TNF-α, and IFNs, and SLE severity^14–21^. However, the serum levels of most of the proinflammatory cytokines measured in this study did not correlate with disease activity indices (SLEDAI and CLASI) or with serum immunological biomarkers, most likely because our study included only SLE patients with LDA. However, it is possible that patients considered to have LDA may express elevated levels of proinflammatory cytokines.

We observed decreases in serum levels of TNF-α, IL-6, VEGF-A, IL-1ra, IL-2, MIP-1α, and IL-8 after add-on HCQ treatment. Our results differ from those of Monzavi et al., who reported no change in serum IL-8 levels after HCQ treatment of newly diagnosed SLE patients^22^. This difference and the observed lack of change in MCP-1 upon HCQ treatment are likely due to the inclusion of only patients with LDA. Blood and urine MCP-1 levels have been reported to be associated with active SLE, particularly lupus nephritis, with urine MCP-1 levels of patients with SLE being significantly higher than those of patients with inactive lupus nephritis and controls^23–25^. In the present study, there was no significant difference in the serum MCP-1 levels before HCQ treatment between patients with and without a history of lupus nephritis. Since the patients included in this study had SLE without any active major organ involvement, it is possible that the serum MCP-1 levels did not significantly decrease after the add-on HCQ treatment. Serum IL-6, IL-8, IL-18, and IFN-α levels have been proposed to be useful predictors of SLE relapse^18,19,26^. We found that HCQ was effective in reducing serum proinflammatory cytokine levels even in patients with LDA, suggesting that HCQ may help prevent relapse of SLE.

Despite its common use, the mechanism of action of HCQ in autoimmune diseases is unclear. HCQ is a weak base known to raise the pH of acidic intracellular vesicles and interfere with their physiological functions, including autophagy and antigen processing^13^. HCQ also interferes with intracellular signaling, which may suppress the response to engagement of the innate Toll-like receptors (TLRs), thereby inhibiting the production and release of cytokines and promoting apoptosis in lymphocytes and endothelial cells^27,28^. Both of these HCQ-induced processes may contribute to the decreased production of proinflammatory cytokines. In addition, HCQ-mediated inhibition of TLR activation suppresses the activity of plasmacytoid dendritic cells and autoreactive B cells in SLE patients^28,29^, leading to a reduction in inflammation. Indeed, Sacre et al. demonstrated that HCQ treatment of SLE patients reduced the ability of plasmacytoid dendritic cells to produce IFN-α and TNF-α in response to TLR-9 and TLR-7 stimulation in vivo^11^. We believe that the HCQ-induced decrease in cytokine observed in the present study may be mediated by these effects.

We previously reported that HCQ modulates the serum levels of S100A8 and S100A9, which are associated with disease activity in SLE patients with LDA^30^. S100 proteins are components of neutrophil extracellular traps (NETs)^31^, which play an important role in the pathogenesis of SLE^32–34^. In addition, chloroquine and HCQ are reported to inhibit NETs in vivo and in vitro^35–37^, suggesting the involvement of S100 protein regulation in their underlying mechanism of action. S100A8 and S100A9 proteins upregulate the expression of proinflammatory cytokines such as IL-6 and IL-8^38–41^; therefore, the HCQ-mediated modulation of S100 proteins may also be involved in the suppression of proinflammatory cytokine expression in SLE patients.

This study has several limitations. First, we did not monitor HCQ adherence by measuring blood HCQ levels. Second, whether the change in proinflammatory cytokine levels were a direct result of add-on HCQ treatment is difficult to determine unequivocally because some patients were receiving other immunosuppressants. Third, the sample size was small due to the strict inclusion and exclusion criteria. This study was an exploratory study and the sample size was not strictly adjusted due to exclusion criteria. We believe that a prospective study with a calculated sample size and a control group is needed to support the results of the present study. Finally, the serum level of IFN-α was undetectable in most patients due to sub-sensitivity. Nevertheless, our study has merit because it is the first to demonstrate the effect of add-on HCQ treatment on proinflammatory cytokines in SLE patients with LDA.

In conclusion, we found that add-on HCQ treatment decreased the serum levels of several proinflammatory cytokines in SLE patients with LDA. Our results suggest that add-on HCQ treatment may reduce proinflammatory cytokine expression in SLE patients. Long-term prospective studies are needed to clarify whether proinflammatory cytokine modulation caused by an add-on HCQ treatment is associated with improved prognosis or prevention of relapse in SLE.

## Patients and methods

### Patients

This single-center exploratory study prospectively enrolled subjects diagnosed with SLE according to the American College of Rheumatology criteria^42^ or the Systemic Lupus Collaborating Clinics criteria^43^. All enrolled patients began HCQ treatment for the first time between September 2015 and March 2019. All patients had a ≥ 3-month history of LDA before enrollment, defined as (i) a SELENA-SLEDAI score ≤ 8 with no activity in major organ systems (renal involvement, neuropsychiatric SLE, cardiopulmonary involvement, or vasculitis); (ii) current prednisolone or equivalent dose of ≤ 10 mg per day; and (iii) well-tolerated maintenance doses of other immunosuppressant. Pregnant women and patients, who were not in complete renal remission^44^, regardless of lupus nephritis history, were excluded. Patients who began anti-thrombotic therapy or add-on immunosuppressants or increased their glucocorticoid dose after starting the HCQ treatment were excluded from the study. Informed consent was obtained from all participants. The study was approved by the ethics committee of Kagawa University (Heisei30-047).

### Treatment and outcomes

Patients were administered oral HCQ sulfate (Plaquenil; Sanofi-Winthrop, Paris, France) continuously for at least 3 months. HCQ was administered at a dose based on the ideal body weight (IBW) calculated using the modified Broca’s method: 200 mg daily for patients with IBW < 46 kg; 200 mg and 400 mg on alternate days for IBW ≥ 46 kg and < 62 kg; and 400 mg daily for IBW ≥ 62 kg. The primary endpoint was the change in proinflammatory cytokine concentrations with additional HCQ administration. Clinical parameters (age, sex, HCQ dose, immunological biomarkers, disease activity indices, and skin scores) were recorded before and after HCQ treatment. Disease activity was evaluated using the SELENA-SLEDAI 2011 criteria^45^. Cutaneous disease activity was evaluated using the CLASI^46^. In accord with the CLASI improvement criteria of Klein et al.^47^, the principal investigator designated the CLASI score as improved, unchanged, or worse compared with the previous visit. Patients classified as improved were defined as ‘CLASI responders,’ and those classified as unchanged or worse were defined as ‘CLASI non-responders.’ Immunological activity was determined by measuring the serum levels of complement factors (C3, C4, and CH50), anti-double stranded DNA (dsDNA) antibodies, and counts of total white blood cells, lymphocytes, and platelets. Complete blood counts were measured using the multi-item automated hematology analyzer XN-3000. The complement factors were measured using the following assay kits: N-assay TIA C3-SH Nittobo, N-assay TIA C4-SH Nittobo, and auto CH50-L “Seiken” II. Anti-double-stranded DNA antibodies were measured using the MESACUP DNA-II test “ds” until October 2017 and the STACIA MEBLux test dsDNA kit since November 2017. The dsDNA antibody assay kit was changed during the study period, but there were no changes in the test kits for the same patients. Specific ELISA kits were used to measure serum IFN-α (Human IFN alpha Platinum ELISA Kit, Thermo Fisher Scientific Inc., Carlsbad, CA, USA) according to the manufacturers’ instructions. Serum TNF-α, IL-2, IL-6, IL-8, VEGF-A, MCP-1, MIP-1α, and IL-1 receptor antagonist (IL-1ra) were measured using a multiplex immunoassay (Luminex Assay, R&D Systems Minneapolis, MN, USA).

### Statistical analysis

Data are presented as the mean ± SD unless otherwise noted. Immunological biomarkers and proinflammatory cytokine levels were compared using paired *t* test for continuous variables or the Wilcoxon signed-rank test for non-normally distributed data. The relationship between the clinical SLE improvement index and the baseline levels of proinflammatory cytokines and the change in proinflammatory cytokine levels before and after HCQ administration was analyzed using the Wilcoxon rank sum (Mann–Whitney *U*) test. The association between the clinical SLE improvement index and the rate of change in proinflammatory cytokines was analyzed using the Mann–Whitney *U* test. The association between pro-inflammatory cytokine levels and clinical variables was determined using correlation analyses (Spearman’s correlation coefficient). All *p* values were two-sided, and *p* < 0.05 was considered significant. Data were analyzed using JMP 15 software (SAS Institute, Cary, NC, USA).

### Ethical approval

The study was approved by the ethics committee of Kagawa University (Heisei30-047) and was prospectively registered. All participants gave their written informed consent before entering the study. The study was conducted in accordance with the Declaration of Helsinki.

## Data Availability

The datasets generated during and/or analyzed during the current study are available from the corresponding author on reasonable request.
